# Biological Patch in the Repair of Rotator Cuff Tears: Functional and Clinical Evaluation of Twenty-Three Cases with a Mean Follow-Up of Six Years

**DOI:** 10.3390/jcm13185596

**Published:** 2024-09-20

**Authors:** Nicolò Nuvoli, Elisa Troiano, Azzurra Masini, Giovanni Battista Colasanti, Nicola Mondanelli, Stefano Giannotti

**Affiliations:** 1 Department of Medicine, Surgery and Neurosciences, University of Siena, Viale Mario Bracci 16, 53100 Siena, Italy; nuvoli.nicolo@gmail.com (N.N.); troiano.elisa@gmail.com (E.T.);; 2 Section of Orthopedics, Azienda Ospedaliero Universitaria Senese, Policlinico Santa Maria alle Scotte, Viale Mario Bracci 16, 53100 Siena, Italy

**Keywords:** rotator cuff repair, shoulder augmentation, porcine dermal patch, human dermal patch, shoulder arthroscopy, rotator cuff augmentation

## Abstract

**Background:** Rotator cuff (RC) repair leads to less than optimal results when dealing with massive lesions, so the use of scaffolds as biological support has been proposed to improve RC repair site biology. The present study aims to evaluate the clinical and radiographical outcomes of a series of patients suffering from massive or irreparable RC tears treated with patch (porcine or human scaffolds) repair (augmentation or bridging). **Methods:** Twenty-three patients with a minimum follow-up of 24 months were subjectively, clinically, and radiographically assessed before and after surgery. Three different patient-related outcome measurements (PROMs) (American Shoulder and Elbow Surgeons score, Constant score, visual analog scale) were used for the subjective and clinical evaluation, while plain radiographs and magnetic resonance imaging where employed for radiographical follow-up. **Results:** Regardless of the technique (augmentation or bridging) or type of scaffold (porcine or human) employed, at follow-up, all patients experienced a statistically significant improvement in all PROMs and clinically. **Conclusions:** Patch repair represents a valid salvage procedure in massive or irreparable RC tears, improving the quality of life and eventually delaying the need for prosthetic replacement.

## 1. Introduction

Rotator cuff (RC) tears affect more than 50% of patients older than 60 years and represent the most common cause of debilitating pain and loss of function of the shoulder [1,2]. A wide variety of surgical options are available for treating massive RC tears, ranging from arthroscopic repair to shoulder arthroplasty [1]. However, even if the employment of reverse shoulder prosthetic replacement has become more common, special attention should be given to the younger population. In fact, reverse shoulder prosthesis may lead to impaired range of motion, a potential need for further revisions, and a significant impact on daily life [1,3]. It is therefore preferable to recur for tears repair in younger people, even in the case of massive lesions. It has been recently demonstrated that this surgical option leads to long-term improvements, and results are superior to the baseline also in the case of repair failure [1]. When a complete repair is not feasible, the use of grafts has been suggested to improve the healing potential. Initially described by Neviaser et al. [4] in 1978, several studies have reported that grafts used in massive and irreparable RC tears can improve tissue quality, promoting biological healing and biomechanical integrity of the repaired tendons, and representing today an accepted method for repair of larger RC tears [5,6,7,8]. Graft materials include autografts, allografts, xenografts, and synthetic or hybrid grafts [9,10]. Biological grafts consisting of bioactive and decellularized membranes with properties such as the tendon extracellular matrix may improve cell proliferation and neoangiogenesis in the repair site, while synthetic grafts may have excellent mechanical resistance but little or no biological properties with possible limitation to the growth of new tendinous tissue, triggering inflammatory responses and foreign body reactions [11,12]. According to the morphology of the RC tear, two different reconstruction methods may be employed. When in the presence of a poor-quality tissue but fully repairable tendon, the patch can be sutured on the bursal side of the reinserted RC to provide both mechanical and biological reinforcement to the primary repair (patch augmentation technique) [10,13]. When the RC lesion cannot be repaired without excessive tension (retracted tears, inadequate tendon excursion), the patch can be interposed between the residual tendon and the tuberosity, bridging the gap (patch bridging technique) [10,14].

With regard to the not-so-satisfactory long-term outcomes of reverse shoulder prostheses in younger patients for massive RC tears, growing attention has been paid to alternative surgical options. In particular, tendon repair and reconstruction have been addressed with patches. However, the existing literature is still scarce, so our work aims to contribute to exploring the topic. The primary aim of the present study was to evaluate the subjective and clinical results at a mid-term follow-up, in terms of functional recovery and shoulder pain, in a series of patients with massive or irreparable RC lesions treated with repair through patch augmentation or bridging using alternatively a commercially available porcine xenograft or a human allograft. The secondary aim was to eventually highlight the possible differences between the two types of grafts and between the two surgical techniques employed. Since there is an increasing interest in rotator cuff repair in order to avoid or, at least delay, shoulder arthroplasty, we hope this study plays a role in expanding the available knowledge on the topic.

## 2. Materials and Methods

*Study design.* A retrospective study was conducted. All surgical reports of patients who underwent RC repair at our institution from 2011 to 2021 were evaluated and are registered in a database (Microsoft Excel, Microsoft^®^, Seattle, WA, USA). Inclusion criteria were as follows: (1) patients undergoing RC repair with patch augmentation or bridging for massive and irreparable RC tears, (2) operated on by a single surgeon (S.G.), (3) with a minimum follow-up of 24 months, (4) who underwent plain radiographs and magnetic resonance imaging (MRI) evaluation both pre-operatively and post-operatively. Exclusion criteria were as follows: (1) patients undergoing superior capsular reconstruction, (2) patients suffering from connective tissue disorders or rheumatic diseases, (3) with a minimum follow-up of less than 24 months, or (4) with incomplete subjective, clinical, or radiological pre-operative and/or post-operative evaluation.

The primary aim of the study was to verify how, using both xenografts and allografts, the use of patches can be effective in assisting the healing of massive and/or irreparable lesions. The secondary goal was to compare the clinical and functional differences between the two different types of grafts and between the two different surgical techniques.

At our institution, no Ethical Committee or Institutional Review Board approval is needed for retrospective studies, and all patients gave their informed consent to data collection and their anonymous use for scientific and teaching purposes. The study was performed in accordance with the ethical standards as laid down in the 1964 Declaration of Helsinki and its later amendments or comparable ethical standards.

*Surgical procedure.* An acellular scaffold of collagen and elastin derived from porcine dermal tissue (Zimmer^®^ Collagen Repair Patch, Zimmer Biomet, Warsaw, IN, USA; collagen) or a human allograft prepared by the Dermal Tissue Bank of our institution (lyophilized de-epidermized decellularized human dermis; DED-LYO) were used, depending on availability. Also, according to the morphology of the lesion and the quality of the tendon, an augmentation or a bridging reconstruction was performed.

All patients were placed in contralateral lateral decubitus; a general or loco-regional anesthesia was used; all surgeries were performed by the senior author. An arthroscopic stage was initially performed to visualize the gleno-humeral joint and the sub-acromial space to confirm and evaluate the type of RC tear. Other associated injuries, such as long head of the biceps degeneration or acromio-clavicular osteoarthritis, were also evaluated. These concomitant lesions eventually underwent treatment, respectively: long head biceps tenotomy and mini-Mumford procedure [15]. Once the RC tear was identified and all associated procedures performed arthroscopically, a lateral mini-open approach was carried out as a second step of the surgical procedure. Bursectomy and selective acromioplasty were performed; the humeral footprint was prepared for the housing of the anchors through an initial debridement and micro-fractures according to the Crimson Duvet technique [16].

**Figure 1:**
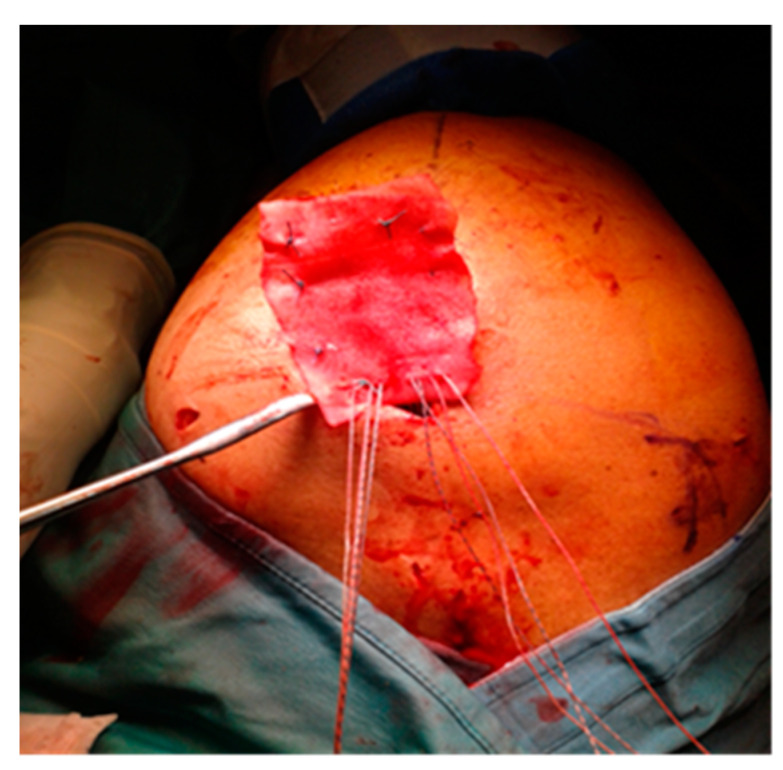
DED-LYO patch augmentation procedure.

**Figure 2:**
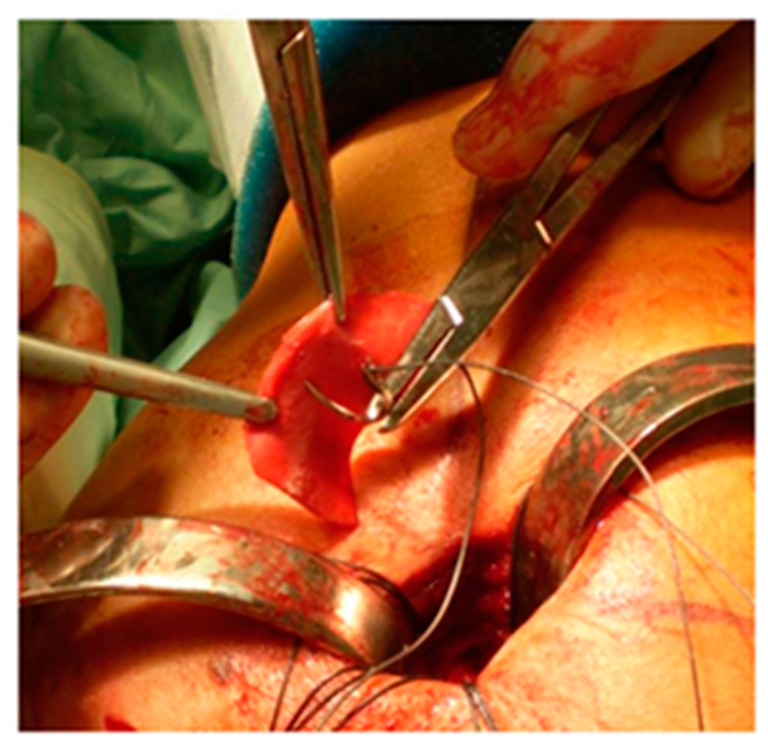
Collagen repair patch bridging procedure.

The edges of the RC tear were identified and debrided; tendon mobilization was attempted to verify if a primary tendon-to-bone repair was possible. When re-insertion to the footprint was possible, but the tendon tissue quality was clinically considered poor, a patch was sutured with non-absorbable stitches on the bursal side of the RC and reinserted together with the tendon onto the respective tuberosity over the anchors (Figure 1). The RC tendon was retracted and non-mobilizable, confirming the impossibility of a direct tendon-to-bone repair sutured to the edges of the tendon stump with non-absorbable sutures and then reinserted into the tuberosity with anchors (Figure 2). In both techniques (augmentation and bridging), and with both the biological implant (xenograft of collagen of porcine origin and DED-LYO allograft), the patch was cut to the appropriate size corresponding to the gap to be reinforced or covered. To control bleeding, in the absence of contraindications, tranexamic acid was administered both intravenously and locally, as previously described [17].

*Post-operative care*. After surgery, to protect the repair, the limb was kept in a brace in 45° of abduction and neutral rotation for 4 weeks. All patients were discharged on the same day or on the first post-operative day, with the possibility of immediately performing assisted passive mobility exercises from 45° of abduction. Assisted active range of motion exercises were started at 4 weeks, and they were implemented at 8 weeks with muscle strengthening exercises, giving priority to the pectoralis major and latissimus dorsi, then to the scapular girdle and to the deltoid muscle. Gradual return to heavy work activities and sporting gestures was allowed at 12 weeks from surgery.

*Clinical and radiological assessment.* Three different patient-related outcome measurements (PROMs) were employed: the American Shoulder and Elbow Surgeons (ASES) score [18], the Constant–Murley Score (CMS) [19], and the visual analog scale (VAS) [20], the latter to quantify the intensity of pain both in the pre-operative and post-operative periods. These PROMs were administered to all patients before surgery (baseline values), at 6 months after surgery, and then annually; the final score was considered the one at the last available follow-up. The difference (Delta) between the baseline and last follow-up values was evaluated. After evaluation, the four groups were compared, and the differences among respective Delta values were assessed. Imaging evaluation (plain radiographs and MRI) was performed to verify centering, coverage of the humeral head and tendon continuity, and signal.

*Statistical analysis.* Fisher’s exact test and the Mann–Whitney U test were used to confirm the homogeneity of the groups by gender and age. The Mann–Whitney test was used to compare the various study groups, and the Wilcoxon test was used to compare variables within the same group. A 2-tailed *p*-value of less than 0.05 was assumed as statistically significant; *p*-values were reported with exact value if significant and with “n.s.” if not significant in text, and with exact value both if significant and not significant in tables. Analysis was performed using a free online software (https://www.socscistatistics.com/, last access 12 September 2024).

## 3. Results

From 2011 to 2021, a total of 42 patients who underwent RC repair with patch augmentation or bridging were identified, but only 23 (55%) completed the minimum follow-up of 24 months, with a thorough subjective clinical and radiological evaluation as indicated, and they were included into the study. The mean follow-up (±standard deviation, SD) was 73 ± 3.2 (range 24–124) months; the mean age at surgery was 59.5 ± 8.3 (range 47–72) years; the male-to-female ratio was 2.3:1 (16 males and 7 females), 16 right and 7 left shoulders. The mean height was 1.7 ± 0.1 (range 1.5–1.85) meters, the mean weight was 72.5 ± 11 (range 50–90) kilograms, and the mean body mass index (BMI) was 24.6 ± 2.2 (range 21.5–29.6) kg/m^2^.

Patients were divided into four groups according to the patch type and the surgical technique employed: group A (collagen augmentation), group B (collagen bridging), group C (DED-LYO augmentation), and group D (DED-LYO bridging). Groups were compared with each other based on the type of patch used (different patch, same surgical technique) and on the type of technique (same patch, different surgical technique).

A total of 13 patients (56.5%) received a porcine-origin patch, and 10 (43.5%) received a human-derived patch; 12 patients (52.1%) were subjected to augmentation for repairable tears with poor-quality tendon, and 11 patients (47.8%) to bridging for irreparable tears. A difference in the mean follow-up regarding the kind of patch employed was observed, with the porcine patch used from 2011 to 2016 and the human patch used from 2017 to 2021. No difference was observed with respect to the surgical technique used (bridging or augmentation). Group A included seven cases, group B six cases, group C five cases, and group D five cases. Details about gender, mean age, and body type are summarized in Table 1. No statistically significant differences among groups were highlighted.

No intra- or post-operative complications, such as adverse reactions to grafts or wound complications, were found. No post-operative and long-term failures such as infections, re-rupture, revision of repair, or subsequent conversion to a reverse shoulder arthroplasty were found.

Comparing the groups to analyze differences between the two types of patches that were used (group A vs. group C and group B vs. group D), no significant differences emerged regarding pre- and post-operative ASES scores between groups (*p* = n.s.). Significant clinical improvements emerged in group C compared to group A with respect to the pre- and post-operative Constant score (mean increase in Constant group C = 65.6 ± 12.3 points, mean increase in Constant group A = 43.8 ± 6.8 points; *p* = 0.023) but not among the group B vs. D (*p* = n.s.). No significant differences emerged regarding pre- and post-operative VAS between groups (*p* = n.s. group A vs. C; *p* = n.s. group B vs. D). Comparing the four groups, with regards to differences between the two techniques that were employed (group A vs. group B, and group C vs. group D), no significant differences emerged regarding pre- and post-operative ASES score (*p* = n.s.), pre- and post-operative Constant score (*p* = n.s.), nor between pre- and post-operative VAS (*p* = n.s.). Results are resumed in Table 2 and Table 3.

**Figure 3:**
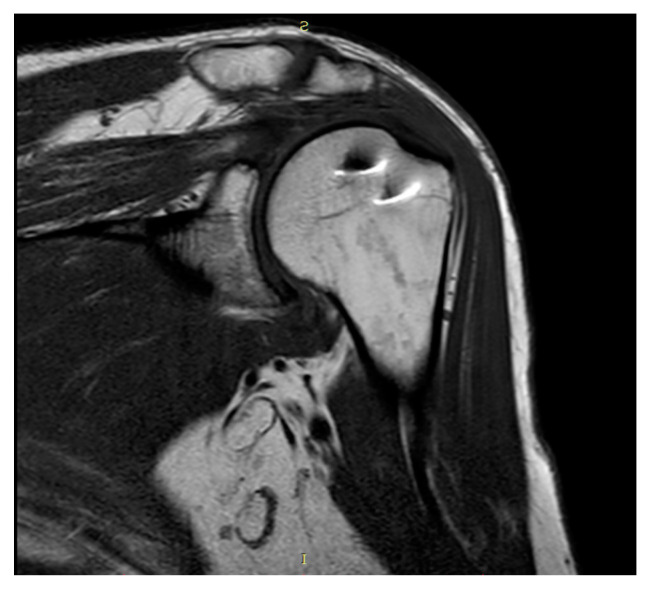
Coronal T1-weighted MRI.

**Figure 4:**
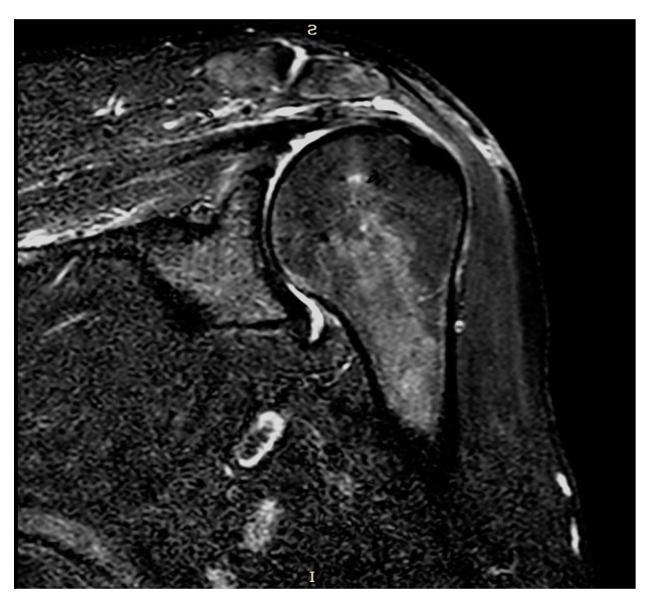
Coronal STIR-weighted MRI.

Plain radiographs and MRI showed coverage and centering of the humeral head, tendon continuity, and absence of fatty degeneration in all patients (Figure 3 and Figure 4).

At the last available follow-up, the mean ASES score increased statistically significantly both for all patients treated with collagen repair (groups A + B) by 48.2 points on average (*p* = 0.002) and for all patients with DED-LYO (groups C + D) by 59.4 points on average (*p* = 0.005). The Constant score also increased significantly, both for the collagen repair groups (A + B) by almost 45 points on average (*p* = 0.001) and for the DED-LYO groups (C + D) by 62.4 points on average (*p* = 0.005). VAS decreased significantly for both patches used: by 6 points on average for the collagen repair groups (A + B) (*p* = 0.001) and by 6.2 points on average for the DED-LYO groups (C + D) (*p* = 0.005). The ASES score also increased statistically significantly both for patients with a patch augmentation (groups A + C) by almost 54 points on average (*p* = 0.002) and for patients with a bridging augmentation (groups B + D) by 52.3 points on average (*p* = 0.003). Also, the Constant score increased significantly both for the augmentation groups (A + C) by almost 53 points on average (*p* = 0.002) and the bridging groups (B + D) by 52 points on average (*p* = 0.003). VAS decreased significantly for both the techniques used: by 6 points for the augmentation groups (A + C) (*p* = 0.002) and by 6.3 points for the bridging groups (B + D) (*p* = 0.003). The baseline and last follow-up values and statistically significant differences are detailed in Table 4.

All patients experienced an important resolution of pain and an increase in shoulder function, with most of them being quite totally able to resume their normal daily life and recreational activities.

## 4. Discussion

RC tears represent a common disease for patients older than 60 years, leading to pain, disability, and eccentric osteoarthritis. Numerous alternative techniques have been proposed [21,22,23,24,25,26,27], but there is still a lack of clear evidence-based guidelines [5,28]. Obviously, treatment should be tailored to the patient, and some authors proposed a treatment algorithm [29]. A recent systematic review emphasizes how RSA for massive RC cuff tears in patients younger than 65 years of age correlated with a higher complication rate compared to RC repair, and a previous RC repair surgery shall not affect the outcome of a subsequent RSA [3]. Primary repair is to be considered the gold standard surgical technique in younger patients; however, it is not always feasible due to both tendon quality and the large extent of the lesions. A proposed method to enhance the healing potential is using grafts on the repair side as augmentation or bridging [4,5,6,7,8].

In our opinion, biological patches may exceed synthetic ones for their ability to form a new tissue with histological, architectural, and mechanical characteristics comparable to those of the native tendon [11,12]. Accordingly, no complications related to the used patches nor adverse reactions were found in our study, and no patient underwent revision surgery or prosthetic replacement. However, patch augmentation is burdened with some potential disadvantages, as described in the literature. Synthetic patches may degrade over time, losing their intrinsic structure, and biological patches may be the cause of immunogenic inflammatory reactions [30]. No cases of RC tendon re-rupture on imaging were highlighted, the tendons showed their continuity and absence of fatty degeneration, and the humeral head appeared centered in the glenoid cavity. There is evidence that patches’ thickness and intrinsic resistance result in pain reduction and allow for an optimized therapeutic rehabilitation process. Furthermore, a lower incidence of retear is guaranteed by the protection and strength of the tendon sutures: patch augmentation resists the superior humeral head migration, reducing the stress on the sutures [30,31]. According to the literature, in the post-operative period, a significant increase in clinical scores in terms of function, muscle strength, and general satisfaction of patients with a significant reduction in pain was found independently of patch type or technique employed. Augmentation and bridging techniques and porcine and human grafts proved to be superimposable and valid in implementing the chances of healing. However, a statistically significant difference in the Constant score between group A and group C was detected, with group C obtaining better results than group A. In our opinion, this difference is mainly due to the presence of a consistent diversity in the mean follow-up between the two groups (103 vs. 32 months) rather than an actual difference inherent to grafts. In fact, the assessment of patients ten years after surgery may have introduced a bias: the progressive advancement of age and the consequent muscle weakening may have been added to the already present shoulder pathology.

The different follow-up period is justified by the fact that from 2017, human dermal allografts were available for clinical use from the local Dermal Tissue Bank. Therefore, preference was given to in-house supplying for economic reasons.

Muscular strength in abduction was evaluated as a part of the Constant score, but abduction is primarily performed by the deltoid muscle and, therefore, is less influenced by rotator cuff muscles. It is known that “strength in abduction” (in pounds) is a major influencer in the final score [32]. Moreover, muscular strength may largely vary among people based on unmodifiable (gender and age, constitution) and modifiable (level of physical activity) parameters [33]. Therefore, evaluating strength might have significance as a difference between the healthy and the affected side more as an absolute value [32]. The goal of treatment is to recover function with respect to the contralateral unaffected side and to the patient’s expectation and pre-lesion level of activity, more than an absolute 100 Constant score.

Limitations are present in the study: the small series, the loss of patients to follow-up, and different follow-up periods in groups C and A, as described above. Also, for future directions, it would be of interest to have the opportunity to take histological samples at second look to test the possible in vivo colonization of the grafts by the host cells. Lastly, a point of interest could be the comparison of the results of patch repair with the results of direct sutures to help clarify the effectiveness of the patch itself. However, in the present retrospective study, two different biological patches in two different configurations (bridging vs. augmentation) in massive and irreparable RC tears were studied. In our practice, if the RC tear is reparable and presents good tissue quality, a patch is not indicated. A comparison between patch repair and “direct repair” falls outside of the aim of the present study, and it would require, in our opinion, a prospective evaluation of the same kind of lesion.

## 5. Conclusions

In the patients evaluated, patch repair proved to be the ideal solution, representing a valid salvage procedure in massive and irreparable RC tears, improving the quality of life and eventually postponing a prosthetic procedure. PROMs demonstrated significant improvement, confirming the durability of both human and porcine patches at mid-term follow-up while also ensuring adequate functionality and resolution of pain. In our opinion, both patches (porcine and human) and both surgical techniques (augmentation and bridging) proved to be effective in the management of massive and irreparable RC tears by reducing painful symptoms and restoring good function.

## Figures and Tables

**Table 1:** Demographic characteristics according to the four groups. The groups are homogeneous according to male–female ratio, mean age, and BMI.

	Group A	Group B	Group C	Group D
**Gender**				
Male	5	4	4	3
Female	2	2	1	2
*p*	0.9			
**Age**				
Mean ± SD	59 ± 10.5	62.6 ± 7.9	61.4 ± 8	54 ± 3.8
Range (min–max)	44–73	50–71	49–69	50–60
*p*	0.4			
**BMI**				
Mean ± SD	25.5 ± 2	24.6 ± 2.8	24.1 ± 1.9	24.1 ± 2.2
Range (min–max)	22.5–27.8	21.9–29.7	21.5–26.2	21.6–27.1
*p*	0.6			

**Table 2:** Functional outcomes expressed in points (mean ± SD).

	Group A	Group B	Group C	Group D
**ASES score**				
Baseline	27.2 ± 12.4	33 ± 20	34.3 ± 22.2	21 ± 15.4
At last follow-up	76.6 ± 13	79.9 ± 20.6	93.9 ± 5	79.9 ± 26
Delta	49.4 ± 7.9	46.8 ± 19.59	59.6 ± 26.1	58.9 ± 31.1
**Constant score**				
Baseline	27 ± 18	35.3 ± 16	28.8 ± 5.4	19 ± 8.2
At last follow-up	70.8 ± 20.4	81.1 ± 20	94.4 ± 7.2	78.2 ± 23.8
Delta	43.8 ± 6.8	46.1 ± 17	65.6 ± 12.3	59.2 ± 23.6
**VAS scale**				
Baseline	8.4 ± 1.5	8.6 ± 1	8 ± 1.8	8.8 ± 1.3
At last follow-up	2.5 ± 1.1	2.3 ± 1.5	2 ± 0.7	2.4 ± 2.4
Delta	−5.8 ± 1.9	−6.3 ± 1	−6 ± 2.1	−6.4 ± 3

**Table 3:** Statistical difference among the Delta values of the four groups. In **bold**, statistically significant differences.

*p*-Value	A vs. C	B vs. D	A vs. B	C vs. D
ASES score	0.913	0.872	0.6672	1
Constant score	**0.0232**	0.5832	0.771	0.528
VAS scale	1	0.857	0.718	0.920

**Table 4:** Clinical improvements expressed in points (mean ± SD) after the operations. In **bold**, statistically significant differences.

	Group A + B	Group C + D	Group A + C	Group B + D
ASES score				
Baseline	29.9 ± 15.8	27.6 ± 19	30.1 ± 16.6	27.5 ± 18.2
At last follow-up	78.1 ±16.3	86.9 ± 19	83.9 ± 13.4	79.9 ± 22
*p*-value	**0.001**	**0.005**	**0.002**	**0.003**
Constant score				
Baseline	30.6 ± 17	23.9 ± 8.3	27.7 ± 14	27.7 ± 15
At last follow-up	75.6 ± 20	86.3 ± 18.6	80.6 ± 19.8	79.8 ± 20
*p*-value	**0.001**	**0.005**	**0.002**	**0.003**
VAS scale				
Baseline	8.5 ±1.2	8.4 ± 1.5	8.2 ± 1.6	8.7 ± 1.1
At last follow-up	2.4 ± 1.2	2.2 ± 1.6	2.3 ± 1	2.3 ± 1.8
*p*-value	**0.001**	**0.005**	**0.002**	**0.003**

## Data Availability

The datasets used and/or analyzed during the current study are available from the corresponding author upon reasonable request.
